# Determinants of self-reported health literacy among older adults in South Korea: a cross-sectional study

**DOI:** 10.3389/fpubh.2025.1628245

**Published:** 2025-09-01

**Authors:** Jieun Shin, Seunghui Baek

**Affiliations:** ^1^Department of Biomedical Informatics, College of Medicine, Konyang University, Daejeon, Republic of Korea; ^2^Department of Health and Exercise Management, Sungshin Women’s University, Seoul, Republic of Korea

**Keywords:** health literacy, older adult, health behavior, logistic regression, South Korea

## Abstract

**Background:**

This study aimed to identify the determinants of self-reported health literacy among older adults aged 65 years and above in South Korea.

**Methods:**

Data were obtained from the 2021 Korean Health Panel Survey, including 4,220 participants aged 65 and above. Statistical analyses such as t-tests, ANOVA, and logistic regression were conducted to examine the effects of personal characteristics and health behaviors on self-reported health literacy.

**Results:**

The analysis revealed that men, younger participants, those with spouses, higher education levels, and individuals engaging in positive health behaviors-including non-smoking, non-drinking, and regular exercise-had significantly higher levels of self-reported health literacy.

**Conclusion:**

These findings suggest the importance of developing targeted strategies to improve health literacy among older adults, particularly focusing on personal characteristics and health behaviors that are associated with higher health literacy.

## 1 Introduction

Health literacy refers to the capacity to access, understand, evaluate, and utilize health-related information to make informed healthcare decisions and maintain overall well-being (1). It plays a pivotal role in promoting effective communication between healthcare providers and patients, supporting adherence to medical recommendations, and empowering individuals to manage chronic conditions successfully.

The adoption and maintenance of healthy lifestyles represent critical determinants of health outcomes, particularly for older adults, who often face challenges navigating complex healthcare systems and medical information. These practices are vital for extending healthspan and enhancing quality of life (1, 2). As individuals age, the ability to interact effectively with the healthcare system becomes increasingly difficult due to cognitive decline, sensory impairments, and the intricacy of medical terminology.

South Korea is among the fastest-aging nations globally, with the proportion of older adults projected to exceed 20% of the total population by 2025 (3). Aging is accompanied by rising rates of chronic illnesses, increased barriers to healthcare access, and escalating medical expenses—factors that collectively underscore the growing importance of health literacy (4). In particular, older adults’ levels of health literacy directly influence their capacity to seek, understand, and use health-related information. Empirical evidence suggests that inadequate health literacy is linked to adverse health outcomes (5, 6).

Health behaviors function as key mediating mechanisms in the relationship between health literacy and health outcomes. Higher health literacy is associated with a greater likelihood of engaging in positive health behaviors, including preventive screenings, regular physical activity, and a nutritious diet. Such behaviors, in turn, contribute to better health outcomes (2, 5).

However, the relationships among health literacy, health behaviors, and health outcomes are unlikely to follow a simple linear pathway. As suggested by previous studies, these variables are influenced by a complex interplay of factors such as social support, access to healthcare, psychological traits, and sociocultural or environmental contexts (2, 7). While health literacy may influence health outcomes through its effect on health behaviors, this pathway is often moderated or mediated by variables such as socioeconomic status, family and community networks, and the complexity of the healthcare system.

Accordingly, this study recognizes the multilayered interactions between health literacy, health behaviors, and health outcomes, and aims to provide a comprehensive analysis of key sociodemographic and behavioral determinants.

Previous research has highlighted that demographic variables such as age, gender, educational attainment, income, and cultural context significantly affect health literacy (5, 7). Given the rapid growth of the older population, disaggregated analysis by age group has become increasingly essential. Studies have also confirmed notable gender-based differences in health behavior, underscoring the necessity of a nuanced analysis of health literacy by both gender and age within the older adults (8).

In addition, behavioral elements such as lifestyle practices, healthcare service utilization, and active health information-seeking behavior have been found to significantly influence health literacy (9). Nonetheless, existing research has largely focused on general determinants of health literacy, with a marked paucity of comprehensive studies centered on older adults in Korea. Therefore, this study aims to examine the levels of health literacy and its associated sociodemographic and behavioral factors among older adults in Korea, using nationally representative data. By doing so, it seeks to lay the groundwork for developing targeted strategies and policies to improve health literacy in this growing population segment.

## 2 Methods

### 2.1 Study design

**Figure 1:**
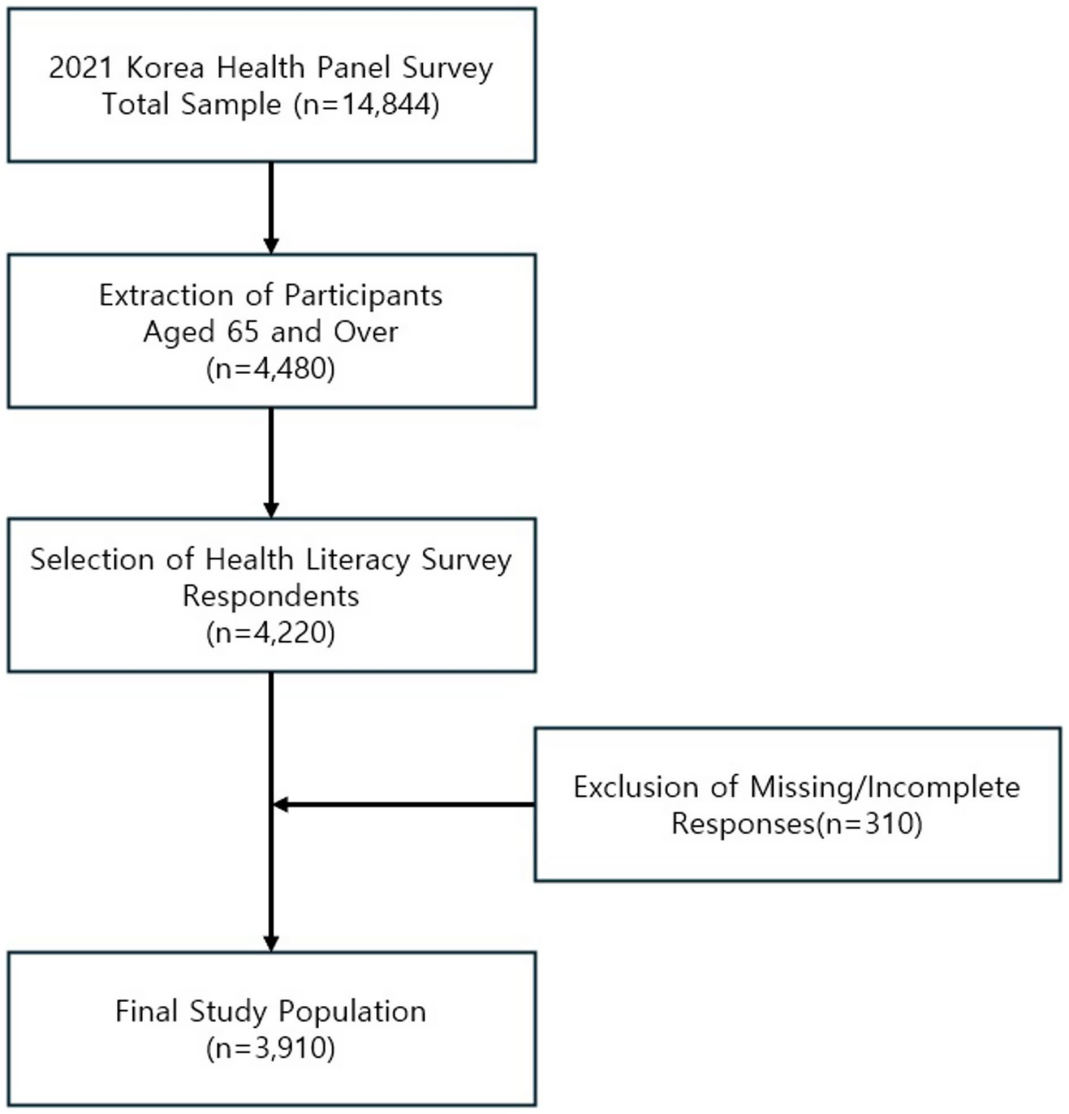
Flowchart of participant selection from the 2021 Korea Health Panel Survey.

This study utilized data from the 2021 Korean Health Panel Survey,^1^ targeting individuals aged 65 years and older. Of the total 4,480 eligible older adults, 260 individuals who did not complete the self-reported health literacy questionnaire were excluded. Additionally, 310 individuals who failed to respond to at least one health behavior question—specifically regarding smoking, alcohol consumption, or physical activity—were also excluded. As a result, a final analytic sample of 3,910 participants was included in the study (Figure 1).

### 2.2 Self-reported health literacy

The Korean version of the HLS-EU-Q16 used in this study was culturally adapted and psychometrically validated for older Korean adults by Chun and Lee (10), through a rigorous process of back-translation and expert review. The validation study reported high internal consistency (Cronbach’s *α* = 0.89), strong item-total correlations (all > 0.40), and a unidimensional factor structure, supporting the construct validity of the tool.

This version was also adopted in the 2021 Korea Health Panel Survey, confirming its applicability in large-scale population-based studies in Korea.

We utilized the Korean version of the HLS-EU-Q16, a 16-item short form of the original HLS-EU-Q47, which was developed for the European Health Literacy Survey to facilitate cross-national comparisons of health literacy levels across European countries (1, 11). For each item, responses were scored as 0 for “very difficult” or “difficult,” and as 1 for “easy” or “very easy.” The total score ranged from 0 to 16, with a cutoff value of 12 commonly used to distinguish levels of health literacy. Scores above 12 were categorized as “adequate” or “high self-reported health literacy,” while scores of 12 or below were categorized as “inadequate” or “low self-reported health literacy” (12).

In this study, participants were classified into high and low health literacy groups based on this 12-point threshold.

### 2.3 General & health-related characteristics

General characteristics included age group (65–69, 70–79, and 80+) and gender (male, female). Health-related characteristics comprised marital status, educational attainment, and economic activity status. Marital status was categorized as “yes” for participants who were currently married and “no” for all others. Educational attainment was grouped into three categories: middle school or less, high school, and college or higher. Economic activity status was defined as “yes” for individuals currently engaged in any form of economic activity and “no” otherwise.

Additional health-related characteristics included smoking, alcohol consumption, and physical activity. Smoking status was coded as “yes” for current smokers (daily or occasional) and “no” for former smokers or those who had never smoked. Alcohol consumption was classified as “no” for individuals who reported never having consumed alcohol in their lifetime or who had not consumed any alcohol in the past year; all other responses were coded as “yes.” Physical activity was assessed based on regular participation in sports or exercise. Participants who reported such activity were categorized as “yes,” and those who did not were categorized as “no.”

### 2.4 Statistics analysis

To analyze differences in self-reported health literacy (HL) levels according to personal characteristics and health behaviors, chi-square tests were performed using IBM SPSS Statistics 25.0. To identify factors associated with HL levels (0 = low, 1 = high), logistic regression analyses stratified by age group (65–69, 70–79, and 80+) and gender (male, female) were conducted. Marital status, educational attainment, and economic activity were included as common covariates. Health behaviors—specifically smoking, alcohol consumption, and physical activity—were entered into the model using the forward selection method. Odds ratios (ORs) and 95% confidence intervals (CIs) were calculated for each variable. A *p*-value of <0.05 was considered statistically significant.

To visualize the distribution of health behavior practices among participants, Sankey diagrams were generated based on the number of healthy behaviors practiced (ranging from 0 to 3), stratified by gender and age group. Health behaviors were defined by three domains: smoking, alcohol consumption, and physical activity. A score of 1 was assigned to each behavior if a participant practiced a healthy behavior (e.g., non-smoking, moderate drinking, or regular physical activity), and 0 otherwise. The total health behavior score was then summed and classified into four categories: 0 (none practiced) to 3 (all practiced).

The visualizations were developed in the Python-based Google Colaboratory environment using the go. Sankey () function from the Plotly library. These Sankey diagrams were used to intuitively depict the flow from the number of healthy behaviors practiced to the level of self-reported HL (high vs. low) across different age and gender subgroups, enabling visual comparisons of patterns in HL and health behavior engagement among older adults.

## 3 Result

### 3.1 Descriptive statistics

When examining respondents’ awareness of the various dimensions of self-reported health literacy (HL) by survey item, the results revealed notable differences across domains (Table 1).

**Table 1:** Response rates by health literacy item.

Health Literacy Item	Response to Health Literacy Item		
	Difficult	Easy	Not sure
find information about treatment for an illness you are worried about	2,807 (71.8)	996 (25.5)	107 (2.7)
know where to get professional help when I am sick	2,420 (61.9)	1,427 (36.5)	63 (1.6)
understand what my doctor has told me	939 (24)	2,950 (75.4)	21 (0.5)
understand the doctor’s or pharmacist’s instructions on how to take prescribed medications	605 (15.5)	3,287 (84.1)	18 (0.5)
determine if I need to see another doctor after seeing my doctor	2,620 (67)	1,127 (28.8)	163 (4.2)
use the information I get from my doctor when making decisions about the treatment of my condition	2,424 (62)	1,358 (34.7)	128 (3.3)
follow directions given to me by my doctor or pharmacist	596 (15.2)	3,304 (84.5)	10 (0.3)
seek information about how to manage mental health problems such as stress or depression	2,655 (67.9)	1,072 (27.4)	183 (4.7)
understand health risk warnings about behaviors such as smoking, lack of exercise, and drinking too much alcohol	1,426 (36.5)	2,390 (61.1)	94 (2.4)
understand why I need a health screening	768 (19.6)	3,113 (79.6)	29 (0.7)
determine if information about health risks from the media is reliable	2,329 (59.6)	1,363 (34.9)	218 (5.6)
decide how to protect myself from illness based on information I get from the media	2,484 (63.5)	1,226 (31.4)	200 (5.1)
identify activities that help my mental health	2,336 (59.7)	1,451 (37.1)	123 (3.1)
understand advice from family and friends about my health	753 (19.3)	3,132 (80.1)	25 (0.6)
understand information from the media about how I can be healthier	1883 (48.2)	1901 (48.6)	126 (3.2)
determine how my daily behaviors relate to my health	2010 (51.4)	1778 (45.5)	122 (3.1)

Respondents were asked to rate the difficulty of each health literacy task as “Difficult,” “Easy,” or “Not sure.” Values are presented as number (%).

Older adults reported greater difficulty in tasks related to information-seeking and evaluation—particularly in finding information about treatment (71.8%), managing mental health problems (67.9%), deciding whether to seek a second opinion (67.0%), and determining the reliability of media-based health information (59.6%). In contrast, HL was relatively high in domains involving direct communication with healthcare providers, such as understanding medication instructions (84.1%), following directions from doctors or pharmacists (84.5%), and understanding advice from family and friends (80.1%).

Sociodemographic and health behavior variables were analyzed across age and gender groups, and all variables demonstrated statistically significant differences (*p* < 0.001) (Table 2). Men were more likely to be married and economically active, while women tended to have lower educational attainment but reported more favorable health behaviors, such as lower rates of smoking and alcohol consumption. Engagement in healthy behaviors declined with age in both genders.

**Table 2:** Results of the distribution of general characteristics and health behaviors by gender and age of subjects.

General characteristics	Age and Sex	65–69 yr			70–79 yr			80–89 yr			All		
Male	Female	All	Male	Female	All	Male	Female	All	Male	Female	All
All	489 (100)	668 (100)	1,157 (100)	950 (100)	1,170 (100)	2,120 (100)	288 (100)	345 (100)	633 (100)	1727 (100)	2,183 (100)	3,910 (100)
Marital Status												
No	65 (13.3)	205 (30.7)	270 (23.3)	118 (12.4)	528 (45.1)	646 (30.5)	35 (12.2)	243 (70.4)	278 (43.9)	218 (12.6)	976 (44.7)	1,194 (30.5)
Yes	424 (86.7)	463 (69.3)	887 (76.7)	832 (87.6)	642 (54.9)	1,474 (69.5)	253 (87.8)	102 (29.6)	355 (56.1)	1,509 (87.4)	1,207 (55.3)	2,716 (69.5)
χ^2^(p)	47.757 (<0.001)			264.729 (<0.001)			216.447 (<0.001)			467.974 (<0.001)		
Education Level												
≤ Middle School	250 (51.1)	472 (70.7)	722 (62.4)	539 (56.7)	969 (82.8)	1,508 (71.1)	171 (59.4)	307 (89)	478 (75.5)	960 (55.6)	1748 (80.1)	2,708 (69.3)
High School	161 (32.9)	159 (23.8)	320 (27.7)	274 (28.8)	166 (14.2)	440 (20.8)	76 (26.4)	29 (8.4)	105 (16.6)	511 (29.6)	354 (16.2)	865 (22.1)
≥ College	78 (16)	37 (5.5)	115 (9.9)	137 (14.4)	35 (3)	172 (8.1)	41 (14.2)	9 (2.6)	50 (7.9)	256 (14.8)	81 (3.7)	337 (8.6)
χ^2^(p)	56.551 (<0.001)			188.813 (<0.001)			75.694 (<0.001)			299.565 (<0.001)		
Economic Activity Status												
No	158 (32.3)	409 (61.2)	567 (49)	461 (48.5)	817 (69.8)	1,278 (60.3)	197 (68.4)	268 (77.7)	465 (73.5)	816 (47.2)	1,494 (68.4)	2,310 (59.1)
Yes	331 (67.7)	259 (38.8)	590 (51)	489 (51.5)	353 (30.2)	842 (39.7)	91 (31.6)	77 (22.3)	168 (26.5)	911 (52.8)	689 (31.6)	1,600 (40.9)
χ^2^(p)	94.467 (<0.001)			99.374 (<0.001)			6.931 (0.008)			179.055 (<0.001)		
Smoking												
Yes	135 (27.6)	10 (1.5)	145 (12.5)	157 (16.5)	20 (1.7)	177 (8.3)	21 (7.3)	4 (1.2)	25 (3.9)	313 (18.1)	34 (1.6)	347 (8.9)
No	354 (72.4)	658 (98.5)	1,012 (87.5)	793 (83.5)	1,150 (98.3)	1943 (91.7)	267 (92.7)	341 (98.8)	608 (96.1)	1,414 (81.9)	2,149 (98.4)	3,563 (91.1)
χ^2^(p)	175.588 (<0.001)			150.423 (<0.001)			15.560 (<0.001)			327.216 (<0.001)		
Alcohol Use												
Yes	432 (88.3)	321 (48.1)	753 (65.1)	821 (86.4)	423 (36.2)	1,244 (58.7)	237 (82.3)	99 (28.7)	336 (53.1)	1,490 (86.3)	843 (38.6)	2,333 (59.7)
No	57 (11.7)	347 (51.9)	404 (34.9)	129 (13.6)	747 (63.8)	876 (41.3)	51 (17.7)	246 (71.3)	297 (46.9)	237 (13.7)	1,340 (61.4)	1,577 (40.3)
χ^2^(p)	201.665 (<0.001)			546.374d (<0.001)			181.044 (<0.001)			910.098 (<0.001)		
Exercise												
No	191 (39.1)	293 (43.9)	484 (41.8)	354 (37.3)	569 (48.6)	923 (43.5)	139 (48.3)	224 (64.9)	363 (57.3)	684 (39.6)	1,086 (49.7)	1770 (45.3)
Yes	298 (60.9)	375 (56.1)	673 (58.2)	596 (62.7)	601 (51.4)	1,197 (56.5)	149 (51.7)	121 (35.1)	270 (42.7)	1,043 (60.4)	1,097 (50.3)	2,140 (54.7)
χ^2^(p)	2.677 (0.102)			27.569 (<0.001)			17.819 (<0.001)			40.028 (<0.001)		
Health Behaviors Count												
0	57 (11.7)	6 (0.9)	63 (5.4)	76 (8)	8 (0.7)	84 (4)	11 (3.8)	1 (0.3)	12 (1.9)	144 (8.3)	15 (0.7)	159 (4.1)
1	177 (36.2)	140 (21)	317 (27.4)	303 (31.9)	193 (16.5)	496 (23.4)	107 (37.2)	57 (16.5)	164 (25.9)	587 (34)	390 (17.9)	977 (25)
2	233 (47.6)	326 (48.8)	559 (48.3)	498 (52.4)	602 (51.5)	1,100 (51.9)	150 (52.1)	210 (60.9)	360 (56.9)	881 (51)	1,138 (52.1)	2019 (51.6)
3	22 (4.5)	196 (29.3)	218 (18.8)	73 (7.7)	367 (31.4)	440 (20.8)	20 (6.9)	77 (22.3)	97 (15.3)	115 (6.7)	640 (29.3)	755 (19.3)
χ^2^(p)	176.488 (<0.001)			176.488 (<0.001)			176.488 (<0.001)			176.488 (<0.001)		

The table presents the distribution of sociodemographic factors (marital status, education level, and economic activity) and health behaviors (smoking, alcohol use, physical activity, and number of health behaviors practiced) among older adults, stratified by age group and gender. Values are expressed as number (%). Statistical significance was assessed using chi-square (χ^2^) tests.

An analysis of sociodemographic characteristics and health behaviors by age and gender revealed statistically significant differences across all variables (*p* < 0.001, Table 2).

In terms of marital status, men were consistently more likely to be married than women across all age groups. Among those aged 65–69, 86.7% of men were married compared to 69.3% of women. This pattern persisted in the 70–79 age group (men: 87.6%, women: 54.9%) and was even more pronounced in the 80–89 age group (men: 87.8%, women: 29.6%). Educational attainment also showed marked gender and age differences. Women had a lower proportion of higher education (college or above) across all age groups. Notably, among women aged 80–89, 89% had completed only middle school or less, compared to 59.4% of men in the same age group. Overall, 80.1% of women and 55.1% of men had an education level of middle school or less. Economic activity was significantly higher among men. In the 65–69 age group, 67.7% of men were economically active, compared to just 38.8% of women. Similar patterns were observed in the 70–79 (men: 54.5%, women: 30.2%) and 80–89 (men: 31.6%, women: 22.3%) age groups. Smoking and alcohol consumption were also substantially higher in men. The overall current smoking rate was 18.2% among men and only 1.6% among women. Similarly, 86.3% of men reported current alcohol consumption, compared to 38.6% of women. Engagement in regular physical activity was generally higher in men. In the 65–69 age group, 60.9% of men and 56.1% of women reported engaging in regular exercise. This trend continued in the 70–79 group (men: 62.7%, women: 51.4%) and the 80–89 group (men: 51.7%, women: 35.1%). Regarding the number of healthy behaviors practiced (based on a 0–3 score for non-smoking, non-drinking, and regular exercise), there were clear gender differences. A higher proportion of women reported practicing all three healthy behaviors compared to men. Specifically, among those aged 65–69, 29.3% of women versus only 4.5% of men practiced all three. This trend continued in the 70–79 group (women: 31.4%, men: 7.7%) and the 80–89 group (women: 22.3%, men: 6.9%).

### 3.2 Self- reported HL levels among older adults

Chi-square analyses revealed significant associations between HL levels and health behaviors in several subgroups (Table 3). For men aged 65–69, the proportion of HL-high individuals was higher among non-drinkers (57.9%) compared to drinkers (36.3%), and among those who exercised (43.0%) compared to those who did not (32.5%). Similar patterns were observed in men aged 70–79 and 80+, where exercise was significantly associated with higher HL. Among women, exercise was significantly linked to HL across all age groups except for those aged 80 and above. Smoking and drinking were generally not significantly associated with HL in women.

**Table 3:** Distribution of self-reported HL levels among older adults.

Age	Behavior	values	Male								Female							
			HL (low)		HL (high)		Total		*χ2*	*p*	HL (low)		HL (high)		Total		*χ2*	*p*
			*n*	(%)	*n*	(%)	*n*	(%)			*n*	(%)	*n*	(%)	*n*	(%)		
65–69	Total		299	(61.1)	190	(38.9)	489	(100)			472	(70.7)	196	(29.3)	668	(100)		
	Smoking	yes	85	(63)	50	(37)	135	(100)	0.259	0.611	9	(90)	1	(10)	10	(100)	1.832	0.176
		no	214	(60.5)	140	(39.5)	354	(100)			463	(70.4)	195	(29.6)	658	(100)		
	Drinking	yes	275	(63.7)	157	(36.3)	432	(100)	9.845	0.002	219	(68.2)	102	(31.8)	321	(100)	1.766	0.184
		no	24	(42.1)	33	(57.9)	57	(100)			253	(72.9)	94	(27.1)	347	(100)		
	Exercise	no	129	(67.5)	62	(32.5)	191	(100)	5.394	0.020	232	(79.2)	61	(20.8)	293	(100)	18.284	0.000
		yes	170	(57)	128	(43)	298	(100)			240	(64)	135	(36)	375	(100)		
	Health behaviors	0	37	(64.9)	20	(35.1)	57	(100)	16.772	0.001	6	(100)	0	(0)	6	(100)	6.021	0.111
		1	122	(68.9)	55	(31.1)	177	(100)			105	(75)	35	(25)	140	(100)		
		2	134	(57.5)	99	(42.5)	233	(100)			232	(71.2)	94	(28.8)	326	(100)		
		3	6	(27.3)	16	(72.7)	22	(100)			129	(65.8)	67	(34.2)	196	(100)		
70–79	Total		723	(76.1)	227	(23.9)	950	(100)			1,020	(87.2)	150	(12.8)	1,170	(100)		
	Smoking	yes	119	(75.8)	38	(24.2)	157	(100)	0.010	0.921	17	(85)	3	(15)	20	(100)	0.086	0.769
		no	604	(76.2)	189	(23.8)	793	(100)			1,003	(87.2)	147	(12.8)	1,150	(100)		
	Drinking	yes	630	(76.7)	191	(23.3)	821	(100)	1.321	0.250	371	(87.7)	52	(12.3)	423	(100)	0.165	0.685
		no	93	(72.1)	36	(27.9)	129	(100)			649	(86.9)	98	(13.1)	747	(100)		
	Exercise	no	292	(82.5)	62	(17.5)	354	(100)	12.632	0.000	529	(93)	40	(7)	569	(100)	33.232	0.000
		yes	431	(72.3)	165	(27.7)	596	(100)			491	(81.7)	110	(18.3)	601	(100)		
	Health behaviors	0	59	(77.6)	17	(22.4)	76	(100)	11.231	0.011	7	(87.5)	1	(12.5)	8	(100)	20.526	0.000
		1	248	(81.8)	55	(18.2)	303	(100)			181	(93.8)	12	(6.2)	193	(100)		
		2	368	(73.9)	130	(26.1)	498	(100)			534	(88.7)	68	(11.3)	602	(100)		
		3	48	(65.8)	25	(34.2)	73	(100)			298	(81.2)	69	(18.8)	367	(100)		
80+	Total		256	(88.9)	32	(11.1)	288	(100)			334	(96.8)	11	(3.2)	345	(100)		
	Smoking	yes	21	(100)	0	(0)	21	(100)	2.831	0.092	4	(100)	0	(0)	4	(100)	0.133	0.715
		no	235	(88)	32	(12)	267	(100)			330	(96.8)	11	(3.2)	341	(100)		
	Drinking	yes	213	(89.9)	24	(10.1)	237	(100)	1.313	0.252	95	(96)	4	(4)	99	(100)	0.327	0.568
		no	43	(84.3)	8	(15.7)	51	(100)			239	(97.2)	7	(2.8)	246	(100)		
	Exercise	no	131	(94.2)	8	(5.8)	139	(100)	7.803	0.005	217	(96.9)	7	(3.1)	224	(100)	0.008	0.927
		yes	125	(83.9)	24	(16.1)	149	(100)			117	(96.7)	4	(3.3)	121	(100)		
	Health behaviors	0	11	(100)	0	(0)	11	(100)	12.632	0.006	1	(100)	0	(0)	1	(100)	0.153	0.985
		1	101	(94.4)	6	(5.6)	107	(100)			55	(96.5)	2	(3.5)	57	(100)		
		2	130	(86.7)	20	(13.3)	150	(100)			203	(96.7)	7	(3.3)	210	(100)		
		3	14	(70)	6	(30)	20	(100)			75	(97.4)	2	(2.6)	77	(100)		

Values are presented as number (%). Chi-square (χ^2^) tests were used to assess differences in HL levels (low vs. high) across smoking, drinking, exercise, and number of health behaviors, stratified by age group and gender.

In both men and women, a consistent trend emerged: individuals who practiced more healthy behaviors (non-smoking, non-drinking, and regular exercise) were more likely to report high HL. This relationship was especially clear among those who practiced all three behaviors, with HL-high rates peaking in these groups, although the pattern was less evident in women aged 80 and above.

### 3.3 Key predictors of self-reported health literacy (HL)

**Figure 2:**
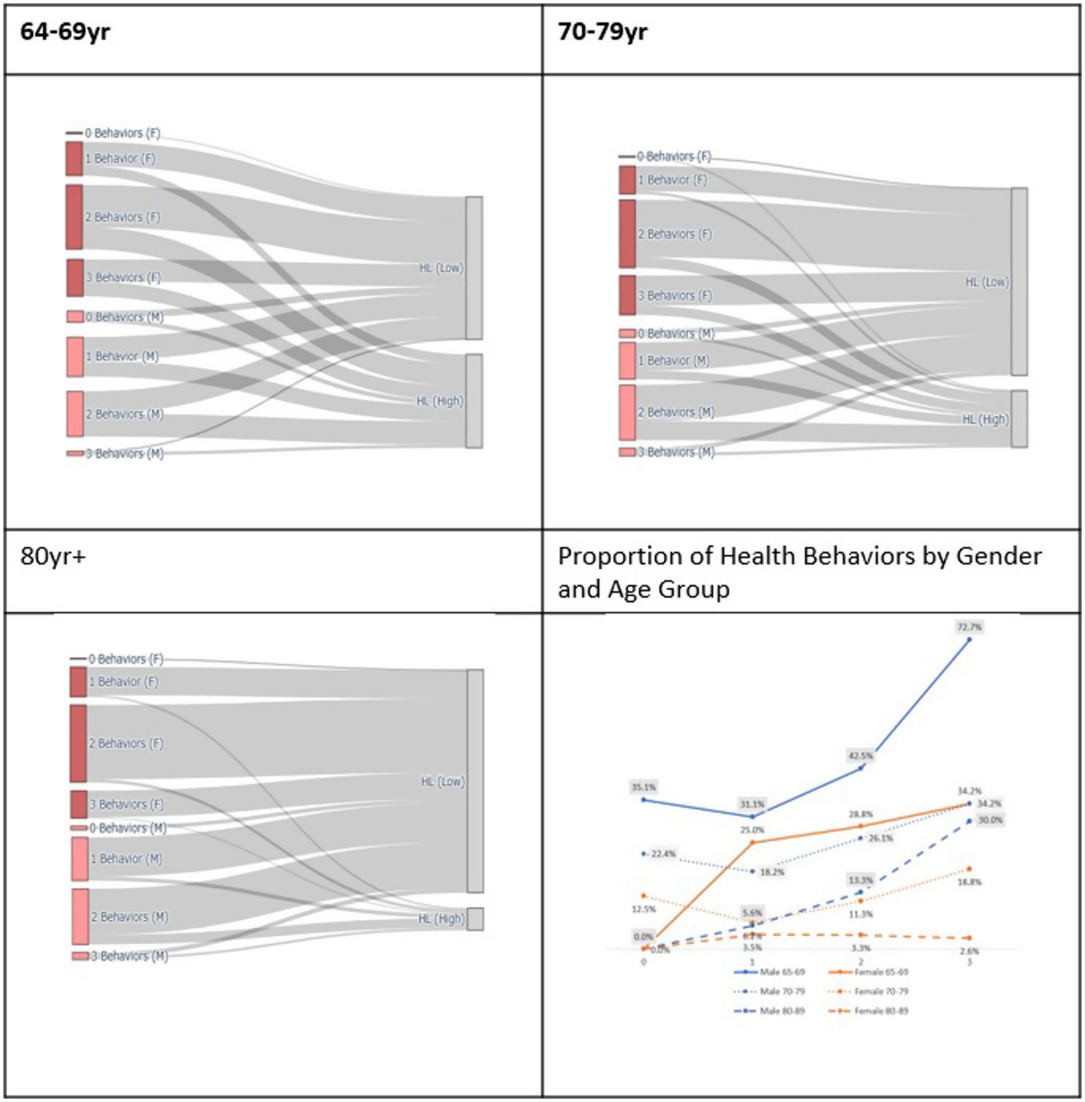
Distribution of health behaviors and health literacy levels by gender and age group.

Logistic regression analyses stratified by age and gender (Table 4; Figure 2) revealed that educational attainment and regular physical activity were consistent predictors of higher self-reported health literacy (HL) across all age and gender groups.

**Table 4:** Predictors of self-reported health literacy by age and gender.

Variable	Male			Female		
	65–69	70–79	80+	65–69	70–79	80+
Marital Status (ref = Unmarried)	1.14 [0.63–2.08]	1.08 [0.66–1.75]	0.93 [0.29–2.98]	1.52 [1.01–2.28]	1.29 [0.87–1.9]	0.44 [0.09–2.21]
Education Level	2.37 [1.81–3.1]	2.19 [1.78–2.69]	1.9 [1.17–3.07]	2.85 [2.13–3.81]	3.44 [2.55–4.65]	4.92 [2.04–11.84]
Economic Activity (ref = No)	1.45 [0.94–2.23]	1.12 [0.81–1.55]	0.6 [0.23–1.56]	1.07 [0.74–1.55]	0.94 [0.61–1.44]	2.12 [0.49–9.23]
Smoking (ref = Yes)						
Drinking (ref = Yes)	2.8 [1.54–5.1]					
Exercise (ref = No)	1.56 [1.04–2.34]	1.47 [1.04–2.08]	2.42 [1.02–5.75]	1.92 [1.32–2.77]	2.44 [1.64–3.63]	

Odds ratios (ORs) and 95% confidence intervals (CIs) are presented. Analyses were stratified by gender and age group. Reference categories are indicated in parentheses.

Among men aged 65–69, those with higher educational attainment were more than twice as likely to report high HL compared to those with lower education (OR = 2.37, 95% CI: 1.81–3.10). In this group, regular physical activity (OR = 1.56, 95% CI: 1.04–2.34) and abstaining from alcohol (OR = 2.80, 95% CI: 1.54–5.11) were also significantly associated with higher HL.

In women aged 65–69, higher educational attainment was also significantly associated with higher HL (OR = 2.85, 95% CI: 2.13–3.81). Additionally, being married (OR = 1.52, 95% CI: 1.01–2.28) and engaging in regular physical activity (OR = 1.92, 95% CI: 1.32–2.77) were positively associated with HL.

In the 70–79 age group, education remained a significant predictor of HL in both men (OR = 2.19, 95% CI: 1.78–2.69) and women (OR = 3.44, 95% CI: 2.55–4.65). Among women, regular physical activity also showed a significant positive association with HL (OR = 2.44, 95% CI: 1.64–3.63). However, this relationship was not observed in men within the same age group.

Among adults aged 80 and older, the relationship between educational attainment and HL was most pronounced in women (OR = 4.92, 95% CI: 2.04–11.84), indicating that highly educated older women were nearly five times more likely to report high HL compared to those with lower education.

In contrast, economic activity, smoking, and marital status (in some age groups) did not show consistent or significant associations with HL across most subgroups.

## 4 Discussion

This study contributes to the growing body of evidence on health literacy (HL) by examining how sociodemographic and behavioral factors influence HL among older adults in Korea. To our knowledge, this is one of the first studies to examine predictors of health literacy among older Korean adults using a nationally representative dataset and a validated tool suitable for international comparisons.

Previous research has established that HL is closely related to demographic characteristics and health-related behaviors (5, 7). The present findings are consistent with and complement these studies by highlighting specific behavioral patterns and subgroup differences. The present findings are consistent with and complement these studies by highlighting specific behavioral patterns and subgroup differences.” In the Korean context, previous research has also identified education, income, and social engagement as significant determinants of HL among older adults (13). Additionally, Jeong and Kim (14) found that over 60% of Korean adults reported inadequate health literacy, which was strongly associated with barriers to health information access such as low education, older age, and digital limitations.

These findings support the relevance of our behavioral variables and affirm the study’s contribution to the national discourse on health literacy determinants.

Notably, regular physical activity was positively associated with HL, aligning with prior studies that suggest health-promoting behaviors enhance both cognitive function and HL (15). Recent research has demonstrated that physical activity improves cognitive capacity, which in turn supports the development of HL (16, 17). This may help explain the positive association between exercise and HL observed in this study. Furthermore, it is plausible that individuals who engage in regular exercise are also more proactive in seeking and processing health information.

Gender differences in HL were also observed, with men reporting higher HL levels than women. This may reflect underlying sociocultural influences or differences in health information–seeking behavior. For example, men are more likely to actively search for health information via digital platforms, whereas women often rely on interpersonal sources such as family or healthcare providers. These behavioral differences can contribute to gender gaps in HL and should be considered when designing targeted interventions, especially in digital health literacy education (18).

While this study did not directly assess digital health literacy, prior research has highlighted its relevance for older adults. For instance, Hwang et al. found that age, gender, education level, and chronic illness significantly affect digital HL among older adults living alone in Korea (19). Such findings suggest that digital access and competency may indirectly influence general HL levels, especially among those with limited exposure to digital health resources. Although this study did not measure digital HL, the absence of significant associations among women aged 80 and older may reflect underlying digital disparities in this group. Future research is needed to clarify the intersection between traditional and digital HL in older populations, particularly those facing technological barriers.

While this study did not directly assess functional ability, previous research has shown that low HL is associated with a greater risk of impairment in activities of daily living (ADLs) among older adults with chronic conditions (20). This suggests that HL may influence not only information processing but also the capacity to perform daily health-related tasks, which should be explored in future research.

Although this study did not examine the relationship between HL and the use of dietary supplements, previous research has shown that older adults with low HL may struggle to understand relevant product information. For instance, Ko et al. reported that individuals with higher HL were better equipped to interpret and apply information about dietary (21). This highlights the importance of improving health communication strategies, particularly among older populations.

Taken together, the findings suggest that HL is influenced by a range of demographic and behavioral factors, and that digital HL, in particular, plays a critical role in maintaining functional health and independent living. Furthermore, the relationship between HL and health behaviors may be bidirectional. Older adults with higher HL are more likely to recognize and modify risky behaviors, such as smoking and alcohol consumption, and are also more inclined to adopt and sustain healthy lifestyles—thereby reinforcing a positive cycle between HL and health behavior (22).

To improve HL among older adults, there is a need for tailored education programs and innovative methods of health information delivery. Digital platforms, in particular, should be leveraged to address the needs of digitally underserved populations. Future research should examine the relationship between traditional and digital HL and develop customized interventions to bridge these gaps.

While previous studies such as Jeong and Kim have emphasized the impact of access barriers on health literacy, they did not explore how specific health behaviors interact with literacy levels (14). Despite the growing body of research on health literacy, few studies have examined its relationship with specific behavioral factors among older Korean adults using nationally representative data.

This study adds to the literature by highlighting age- and gender-specific predictors of HL and by using a validated instrument applied in a large-scale national survey.

Finally, this study used a self-reported measure of HL, which may be subject to response bias. In addition, the generalizability of findings may be limited due to sample characteristics. These limitations should be addressed in future studies employing more diverse measurement tools and representative samples.

## 5 Conclusion

This study investigated key sociodemographic and behavioral determinants of health literacy (HL) among older adults in Korea, using nationally representative data and a validated international tool. Higher HL levels were associated with being male, younger in age, having higher educational attainment, being married, and engaging in regular exercise. These findings offer novel insights into subgroup-specific disparities in HL and reinforce the need for targeted policy interventions.

To improve HL in this population, it is essential to promote physical activity and support the rebuilding of social networks and participation in economic activities—particularly among older women. Tailored HL strategies that consider gender- and age-specific characteristics are crucial.

Future research should focus on developing and evaluating integrated interventions that incorporate digital health literacy, which will be instrumental in informing national policies and designing effective programs to enhance HL among older adults in South Korea.

However, as this study employed a cross-sectional design, causal relationships between health literacy and the associated factors cannot be confirmed. Future studies using longitudinal designs are needed to validate these associations over time.

## Data Availability

The datasets presented in this study can be found in online repositories. The names of the repository/repositories and accession number(s) can be found in the article/supplementary material.

## Footnotes

^1^ https://www.khp.re.kr:444/
